# Enhancing Anticancer Efficacy of Chemotherapeutics Using Targeting Ligand-Functionalized Synthetic Antigen Receptor-Mesenchymal Stem Cells

**DOI:** 10.3390/pharmaceutics15061742

**Published:** 2023-06-15

**Authors:** Susheel Kumar Nethi, Xiaolei Li, Shubhmita Bhatnagar, Swayam Prabha

**Affiliations:** 1 Fels Cancer Institute for Personalized Medicine, Lewis-Katz School of Medicine, Temple University, Philadelphia, PA 19140, USA; snethi@iastate.edu (S.K.N.); xiaolei.li@temple.edu (X.L.); 2 School of Pharmacy, Temple University, Philadelphia, PA 19140, USA; sbhatnagar@temple.edu; 3 Department of Cancer and Cellular Biology, Lewis Katz School of Medicine, Temple University, Philadelphia, PA 19140, USA; 4 Molecular Therapeutics Program, Fox Chase Cancer Center, Temple University, Philadelphia, PA 19111, USA

**Keywords:** cell-based systems, tumor targeting, ligand functionalization, drug delivery, chemotherapy, lung cancer

## Abstract

Mesenchymal stem cells (MSCs) have been studied for their potential in facilitating tumor-targeted delivery of chemotherapeutics due to their tumor-homing characteristics. We hypothesized that targeting effectiveness of MSCs can be further enhanced by incorporating tumor-targeting ligands on MSC surfaces that will allow for enhanced arrest and binding within the tumor tissue. We utilized a unique strategy of modifying MSCs with synthetic antigen receptors (SARs), targeting specific antigens overexpressed on cancer cells. MSCs were surface-functionalized by first incorporating recombinant protein G (PG) on the surface, followed by binding of the targeting antibody to the PG handle. We functionalized MSCs with antibodies targeting a tyrosine kinase transmembrane receptor protein, epidermal growth factor receptor (EGFR), overexpressed in non-small-cell lung cancer (NSCLC). The efficacy of MSCs functionalized with anti-EGFR antibodies (cetuximab and D8) was determined in murine models of NSCLC. Cetuximab-functionalized MSCs demonstrated improved binding to EGFR protein and to EGFR overexpressing A549 lung adenocarcinoma cells. Further, cetuximab-functionalized MSCs loaded with paclitaxel nanoparticles were efficient in slowing orthotopic A549 tumor growth and improving the overall survival relative to that of other controls. Biodistribution studies revealed a six-fold higher retention of EGFR-targeted MSCs than non-targeted MSCs. Based on these results, we conclude that targeting ligand functionalization could be used to enhance the concentration of therapeutic MSC constructs at the tumor tissue and to achieve improved antitumor response.

## 1. Introduction

Tumors exhibit uneven vascular perfusion; the peripheral or rim regions often have nearly normal blood flow while the cells in the core regions of the tumor are often exposed to hypoxic (low-oxygen) conditions [1,2,3]. Tumors also face challenges related to elevated interstitial fluid pressure and a rigid extracellular matrix, which can hinder the transport of solutes within the tumor microenvironment [4]. These pathophysiologic characteristics limit drug delivery and hinder the effectiveness of treatments [5,6,7]. Cell-based drug delivery systems have shown promise in overcoming the critical tissue barriers presented by elevated interstitial fluid pressure and a rigid extracellular matrix within tumors. These systems utilize living cells as carriers to transport therapeutic agents into the tumor microenvironment, enabling active penetration of the tumor stroma [8,9,10]. Multiple cell types, including immune cells [11], macrophages [12], modified tumor cells, and mesenchymal stem cells (MSCs), have been investigated for tumor-targeted drug delivery [13].

Our laboratory has functionalized MSCs with therapeutic nano carriers, enabling their application to tumor-targeted small molecule drug delivery [14,15,16,17,18]. We demonstrated that nanoengineered MSCs effectively accumulate in the tumor stroma and release their small molecule payload, resulting in improved therapeutic efficacy. The goal of the current study was to investigate whether tumor accumulation and retention of MSCs could be further enhanced by functionalizing MSC surfaces with tumor-targeting ligands. Similar to the concept of chimeric antigen receptor (CAR)-T cells [19,20], we carried out synthetic modification of MSCs with an antibody (Ab) targeting specific antigens overexpressed on cancer cells. In this unique approach, recombinant protein G (PG) was successfully incorporated on the surface of MSCs, followed by binding to a full-length Ab (IgG) [21]. Because protein G binds to the Fc region of IgG, antigen-binding affinity of the Ab is expected to be conserved [22].

To test the concept of what we term Synthetic Antigen Receptor (SAR)-MSCs, we functionalized MSCs with Ab, targeting the epidermal growth factor receptor (EGFR), whose overexpression is implicated in the pathogenesis of several malignancies, including non-small-cell lung cancer (NSCLC) [23]. Further, EGFR overexpression is associated with low chemosensitivity, poor prognosis, and reduced survival [24,25]. Cetuximab (Cmab) is an anti-EGFR monoclonal Ab used to treat metastatic lung cancer. It has been extensively investigated in combination with chemotherapy as a standard of care for NSCLC [26]. In the present study, we investigated the comparative therapeutic efficacy of Cmab-functionalized, paclitaxel-loaded SAR-MSCs relative to non-targeted MSCs. Our preclinical studies in NSCLC models demonstrate the potential for improved tumor targeting with EGFR-targeted SAR-MSCs.

## 2. Materials and Methods

### 2.1. Materials

Poly (DL–lactide–co–glycolide) (50:50) (PLGA) (inherent viscosity of 0.55–0.75 dL/g) ester, was obtained from Lactel Absorbable Polymers (Birmingham, AL, USA). Paclitaxel was obtained from TCI America (Portland, OR, USA). Ammonium acetate, chloroform, polyvinyl alcohol, MTS (tetrazolium compound), PMS (phenazine methosulfate), and palmitic acid (PA) were procured from Sigma (St. Louis, MO, USA). Penicillin/streptomycin was purchased from Bioexpress (Kaysville, UT, USA). Dulbecco’s phosphate-buffered saline (DPBS), RPMI 1640, and trypsin-EDTA solution were procured from Life Technologies (Grand Island, NY, USA). Fetal bovine serum (FBS) from Atlanta Biologicals Inc. (Flowery Branch, GA, USA) was used. Recombinant protein G and recombinant human EGFR protein were purchased from Thermo Fisher Scientific (Waltham, MA, USA). Alexa flour 647-labeled control isotype IgG Ab was purchased from Life Technologies (Carlsbad, CA, USA). Cetuximab (Erbitux) was obtained from Eli Lilly and Company (Indiana, IN, USA). Anti-EGFR Ab (D-8) was purchased from Santa Cruz Biotechnology (Dallas, TX, USA). D-Luciferin potassium salt was procured from Gold Biotechnology (Saint Louis, MO, USA). Bone-marrow-derived human mesenchymal stem cells and mesenchymal stem cell growth medium were procured from ScienCell Research Laboratories (Carlsbad, CA, USA). A549-luc-C8 (A549-luc), a firefly luciferase-expressing human lung carcinoma cell line, was purchased from Caliper Lifesciences (Hopkinton, MA, USA).

### 2.2. Derivatization of Protein G with N-Hydroxysuccinimide (NHS) Ester of Palmitic Acid

Recombinant protein G (PG) was reacted with NHS ester of palmitic acid (PA). Briefly, PG solution (1 mg/mL) was prepared in phosphate-buffered saline (PBS) and kept at 37 °C with gentle stirring. NHS ester of PA was dissolved in ethanol (10 mg/mL) and heated to 50 °C for 30 min. The PA solution (10 µL) was added to the PG solution (1 mL) and stirring was continued for 24 h at 37 °C. Palmitic-acid-derivatized protein G (PA-PG) was purified on a Sephadex G-25 column to remove the unreacted lipid, as reported previously [27].

#### Characterization of PA-PG

Purified PA-PG was characterized using ultra-performance liquid chromatography–mass spectrometry (UPLC-MS) to determine the derivatization ratio (number of PA molecules per PG molecule). Liquid chromatography–mass spectrometry (LC-MS) analysis using a Waters Acquity UPLC system coupled to a Waters Synapt G2 HDMS quadrupole orthogonal acceleration time of flight (Q-TOF) mass spectrometer equipped with an LC Column (Waters Acquity UPLC Protein BEH C4, 2.1 mm × 100 mm; particles diameter: 1.7 μm, 35 °C) was performed as described previously [28]. The system was run at linear gradient elution using mobile phase (Phase A: Water containing 0.1% formic acid and Phase B: Acetonitrile containing 0.1% formic acid) at a flow rate of 0.4 mL/min. One mass spectrum (0.2 s scan, *m*/*z* 50–1200) was acquired every 10 s. Three lockspray *m*/*z* measurements of protonated leucine enkephalin were averaged and mass correction applied to the measured *m*/*z* values.

### 2.3. Cell Culture

MSCs, A549-luc cells, and LL/2-luc cells were grown in MSC complete medium, RPMI 1640 medium. and Dulbecco’s modified Eagle’s medium (DMEM) containing 10% *v*/*v* FBS and 1% *v*/*v* penicillin–streptomycin antibiotic solution, respectively. All the cell lines were maintained in a controlled humidity chamber (5% carbon dioxide; 37 °C). All our cell culture studies were performed under these conditions, unless specified otherwise.

### 2.4. Optimization of PA-PG Incorporation on MSC Membrane

#### 2.4.1. Effect of PA-PG Concentration on Cell Viability

MSCs (8 × 10^3^ cells per well) were incubated with different concentrations of PA-PG-containing media (10, 20, 50, and 100 µg/mL) for 24 h and the viability of cells was analyzed using MTS assay. The PA-PG-containing media was replaced with MTS/PMS solution in media and incubated for 1 h at 37 °C. The absorbance was recorded at 490 nm using a microplate reader (Spectramax, Molecular Devices, San Jose, CA, USA). Untreated MSCs and MSCs incubated with PG (50 and 100 µg/mL) served as controls.

#### 2.4.2. PA-PG Coating on MSC Surface

Flow cytometry was used to confirm the binding of Ab to PA-PG-coated MSCs. MSCs in culture were harvested via trypsinization and counted. About 2.5 × 10^5^ cells were incubated with PA-PG (20–100 µg/mL) in a 1.5 mL Eppendorf tube for 1 h at 37 °C under gentle rotation. The cells were washed twice with DPBS and collected using centrifugation at 1000 rpm for 5 min. The cells were then incubated with Alexa Flour 647-labeled IgG (AF647-IgG) for 1 h at 4 °C under gentle rotation. Following incubation, the cells were washed with DPBS by centrifugation as before and the final pellet was resuspended in flow buffer. Binding of the Ab to MSCs was determined by measuring the mean fluorescence intensity of AF647-IgG-labeled MSCs using a BD LSR II flow cytometer.

The incubation time and temperature of the PA-PG coating reaction were optimized by treating MSCs with PA-PG for different durations (0.5, 1, 2, and 3 h) and at different temperatures (4 °C and 37 °C), followed by multiple washings using DPBS. Cells were then incubated with AF647-IgG for 1 h as described above. Ab binding to MSCs was determined via flow cytometry as above.

### 2.5. Ab Incorporation on PA-PG-Coated MSCs

#### 2.5.1. Analysis of Ab Binding to PA-PG-Coated MSCs via Flow Cytometry

Cmab and an isotype control IgG were labeled with IR800 dye (LI-COR Biosciences, Lincoln, NE, USA) using established procedures. MSCs were incubated with PA-PG (50 µg/mL) in serum-free media for 1 h at 37 °C. Labeled Ab (either Cmab or isotype Ig) was added to PA-PG-coated MSCs and incubated for 1 h at 4 °C. Post-incubation, the cells were washed with DPBS, resuspended in flow buffer, and analyzed for IR800 fluorescence intensity via flow cytometry.

#### 2.5.2. Quantitative Analysis of Ab Binding to PA-PG-MSCs

The amount of Ab bound to the surface of PA-PG-coated MSCs was determined using a standard curve constructed using known concentrations of IgG-AF647 Ab (0.01, 0.1, 0.25, 0.5, 1, and 10 µg/mL). AF647 IgG-labeled PA-PG-MSCs were prepared as described above. Labeled MSCs (1 × 10^6^) were then suspended in PBS or a mixture of RIPA buffer and PBS. Fluorescence intensity of the cell suspension was measured using a plate reader (Spectramax), and the concentration of IgG bound to MSCs was calculated from the standard plot.

#### 2.5.3. Qualitative Analysis of Ab Binding to PA-PG-MSCs via Confocal Microscopy

MSCs stably expressing green fluorescent protein were seeded on pre-sterilized 100 mm coverslips in a 6-well plate (50,000 cells/well). One day later, MSCs were incubated with PA-PG (50 µg/mL) in serum-free media for 1 h at 37 °C. The cells were then washed twice with DPBS and then incubated with AF647-labeled Ab (100 µg/mL) for 1 h at 4 °C. The cells were then washed, labeled with Hoechst 33342 dye (to stain the nuclei), and fixed using 4% paraformaldehyde for 15 min at room temperature. The cells were then washed, and the coverslips were mounted on microscopy slides using mounting media (Thermo Fisher Scientific). The slides were then imaged for GFP (MSC), Hoechst (nuclei), and AF647 (Ab) fluorescence using a confocal microscope (Leica, Wetzlar, Germany).

### 2.6. In Vitro Binding of Cmab-MSCs to Its Target

#### 2.6.1. Binding to Immobilized EGFR

Recombinant EGFR protein, dissolved in DPBS (10 µg/mL), was added to 96-well plates, incubated for 2 h at 37 °C, and then washed thrice with DPBS to remove any unbound protein. Non-specific interactions were blocked by incubating the plates with 1% bovine serum albumin in DPBS for 30 min at 37 °C. Unmodified MSCs and Cmab-MSCs were first incubated with calcein AM solution (2 µg/mL) for 1 h and then added to EGFR-coated wells (10,000 cells per well) and incubated for 30 min at 37 °C. The wells were then washed thrice with DPBS to remove unbound and loosely bound cells. The fluorescence intensity of EGFR-bound cells was measured using Spectramax at excitation and emission wavelengths of 485 and 520 nm, respectively.

#### 2.6.2. Binding to A549 Cells

Binding of Cmab-MSCs to A549 cells was evaluated using flow cytometry. First, 3 × 10^5^ A549 cells/well were cultured in multi-well plate overnight followed by incubation with Hoechst dye for 10 min in the dark at 25 °C and washed with DPBS. PA-PG-coated MSCs, control IgG-MSCs, and Cmab-MSCs were first stained with the cell membrane labeling dye CellVue 780. Labeled MSCs (1 × 10^5^ cells/well) were incubated with A549 cells for 30 min at 37 °C. The plates were washed thrice to remove unbound cells. A549 cells, along with the MSCs attached to them, were harvested by trypsinization, washed, and resuspended in a flow buffer. The cells were then analyzed using flow cytometry (BD LSR-II) to determine doublet cells positive for both CellVue780 (ex:744 nm/em:771 nm) and Hoechst (ex:361 nm/em:497 nm) dyes.

### 2.7. Paclitaxel Loaded Cmab-MSCs

#### 2.7.1. Preparation and Characterization of Paclitaxel-Loaded Poly(lactide–co–glycolide) (PLGA) Nanoparticles

Paclitaxel-loaded PLGA nanoparticles were prepared via the emulsion-solvent evaporation method as reported previously [29]. PLGA (32 mg) and paclitaxel (8 mg) were dissolved in 1 mL of chloroform. The oil-in-water emulsion was obtained by mixing the polymer–drug solution with 8 mL of PVA solution (2.5% *w*/*v*) using a probe sonicator (Model W-375, Heat Systems Ultrasonics Inc., Farmingdale, NY, USA) for 5 min at 20 W and stirring overnight at room temperature. The resultant dispersion was placed under vacuum for 1 h to remove chloroform. The PLGA nanoparticles were collected via ultracentrifugation (35,000 rpm; 35 min at 4 °C) (Optima XPN-80 Ultracentrifuge, Rotor type: 50.2 Ti, Beckman Coulter, Brea, CA, USA). The nanoparticles were washed two times using distilled water to remove unentrapped drug and PVA. Final nanoparticles dispersion was centrifuged at 1000 rpm for 5 min to remove aggregates. The dried lyophilized (Labconco, FreeZone 4.5, Kansas City, MO, USA) nanoparticles were stored at −20 °C.

The nanoparticles were characterized using a Delsa Nano C particle analyzer (Beckman Coulter, CA, USA). Nanoparticles dispersion (n = 3) in deionized water (0.1 mg/mL) was used for particle size analysis.

To determine paclitaxel loading, the drug was extracted from methanolic dispersion of nanoparticles (1 mg/mL) overnight using a rotary shaker under gentle agitation and analyzed using reverse-phase high-performance liquid chromatography (HPLC) as reported previously [16]. Drug loading and entrapment efficiency were determined using the following equations:

$$\mathrm{Entrapment}\;\mathrm{efficiency}\;(\%)=\frac{Drug\;amount\;in\;nanoparticles}{Total\;amount\;of\;drug}\times 100$$

$$\mathrm{Drug}\;\mathrm{loading}\;(\%)=\frac{Drug\;amount\;in\;nanoparticles}{Amount\;of\;drug\;loaded\;nanoparticles}\times 100$$

#### 2.7.2. Preparation of Paclitaxel-Loaded Cmab-MSCs

MSCs (2.5 × 10^5^ cells/mL) were incubated with paclitaxel nanoparticles (0.1 mg/mL) dispersion for 4 h at 37 °C under periodic shaking followed by incorporation of either Cmab or isotype control IgG on the surface as described previously. MTS assay was used to study the effect of paclitaxel loading on MSC viability. To visualize nanoparticle-loaded, Cmab-functionalized MSCs, MSCs were incubated with 6-coumarin-loaded nanoparticles followed by Cmab functionalization. Cells were imaged using a confocal microscope to visualize green (6-coumarin nanoparticles) and red (AF647-labeled Cmab) fluorescence.

#### 2.7.3. Impact of Paclitaxel Loading on In Vitro Migration

In vitro migration of paclitaxel-loaded Cmab-MSCs was determined using a trans-well assay. Untreated MSCs, Cmab-MSCs, paclitaxel-loaded MSCs, and paclitaxel-loaded IgG-MSCs were used as controls. MSCs were serum-starved for 24 h prior to the assay. Migration towards either serum-free media, 5% serum-containing media, or tumor-conditioned media (5% serum-containing media with A549 cells for 24 h) was assessed at 37 °C for 20 h. To quantitate the cell migration, cells in the bottom wells were stained using Calcein AM and the percentage of cells migrated was determined using a fluorescence plate reader (Spectramax).

### 2.8. In Vivo Tumor Retention and Anti-Tumor Efficacy Study of Cmab-MSCs

#### 2.8.1. Orthotopic Lung Tumor Models

Animal protocols (University of Minnesota and Temple University) approved by the respective Institutional Animal Care and Use Committees (IACUC) were used. Female SCID Beige mice (4- to 6- weeks old) and female C57BL/6J albino mice (aged 6–8 weeks) were purchased from Charles River Laboratories. An orthotopic human lung tumors were established via intravenous (tail vein) injection of A549-luc cells (1 million) in SCID Beige mice. Likewise, a syngeneic orthotopic mouse lung tumor model was established via tail vein injection of LL/2-luc cells (1 million) in C57BL/6 immunocompetent mice. To image tumor progression, D-luciferin potassium salt solution in DPBS was administered intraperitoneally (150 mg/kg) and mice were imaged using an IVIS Spectrum animal imager (PerkinElmer, Waltham, MA, USA).

#### 2.8.2. Tumor Retention

LL/2-luc lung tumor model was used to determine tumor retention of EGFR-targeted MSCs manufactured using murine anti-EGFR monoclonal antibody (D8). Mice were randomized when tumor bioluminescence reached 5 × 10^6^ photons/s into two groups and injected with 1 million unmodified MSCs or D8-MSCs. Mice were euthanized at 1-week and 2-week intervals. Lung tumor tissues were preserved in RNA-later solution at −20 °C. Further, the stored lung tumors were homogenized using a tissue homogenizer and the total RNA extracted was reverse-transcribed using the cDNA Reverse Transcription Kit (Applied Biosystems, Waltham, MA, USA). Primers targeting THY1 (Hs06633377_s1) and RPLP0 (Hs02786624_g1) were used for qPCR analysis. Real-time PCR was run in quadruplicate using a QuantStudio 12K Flex detection system (Applied Biosystems, Waltham, MA, USA) according to the recommended cycle as described previously [30].

#### 2.8.3. Anticancer Efficacy of Paclitaxel-Loaded EGFR-Targeted MSCs

An initial pilot study was performed to determine the optimal dosing regimen for Cmab-MSCs. Mice bearing A549 lung tumors (~5 × 10^6^ photons/s bioluminescence) were divided randomly into two groups and injected intravenously with 1 × 10^6^ cells/animal with either paclitaxel-loaded IgG-MSCs or paclitaxel-loaded Cmab-MSCs suspended in 200 µL DPBS, once every two weeks.

Following the pilot study, we determined the comparative therapeutic efficacy of paclitaxel-loaded Cmab-MSCs relative to that of paclitaxel-loaded, unfunctionalized MSCs. Saline, Cmab (i.p. administration; 1.1 µg in 200 µL DPBS/mouse), and paclitaxel-loaded IgG-MSCs were used as additional controls. All the treatments were administered bi-weekly, and bioluminescence measurements were performed to follow tumor progression.

### 2.9. Statistical Analysis

All the data are presented as mean ± standard deviation or mean ± standard error of mean. Population distribution was tested for normality using D’Agostino and Pearson test and Kolmogorov–Smirnov test (GraphPad Prism 8). The significance (*p* < 0.05) of observed differences between different groups was determined using Student’s *t*-test or ANOVA. Multiple comparisons between individual treatments was performed using Tukey’s method. Survival analysis among different treatment groups was performed using Log-rank test.

## 3. Results and Discussions

MSCs are a type of adult stem cell that can be isolated from various tissues, including bone marrow, adipose tissue, and umbilical cord tissue [31,32]. MSCs have unlimited potential to differentiate into different cell types depending upon the origin [33]. MSCs play a key role in tissue repair and regeneration [34]. MSCs respond to tissue injury and the consequent release of inflammatory signals by actively migrating to the site of tissue injury [35]. Previous studies show that SDF1- CXCR4/CXCR7 signaling plays a key role in mediating MSC chemotaxis [36]. Tumors co-opt this signaling pathway, resulting in active recruitment of MSCs to the tumor microenvironment, where they can promote immunosuppression [37].

This tumor-homing property of MSCs has been leveraged by several groups to deliver various therapeutic agents to the tumor [35]. MSCs stably transduced with viral and non-viral vectors have been used to deliver cytokines [38], suicidal genes [39,40,41], and antibodies [42] to the tumor. Several investigators, including our group, have engineered MSCs to deliver cytotoxic drugs to the tumor tissue [14,15,16,18,29,43,44,45]. These studies show that the extent of tumor homing and retention of MSCs is a critical determinant of their therapeutic effectiveness.

In the present study, we hypothesized that the tumor-targeting effectiveness of MSCs can be significantly improved by incorporating tumor-targeting ligands on the MSC surface that will allow for enhanced arrest and binding of MSCs within the tumor tissue. We used EGFR as a model tumor antigen because EGFR overexpression has been reported in NSCLC [23,46]. Two different Abs, one specific to human EGFR (Cmab) and another that recognizes mouse EGFR (D8), were used as targeting ligands.

**Figure 1:**
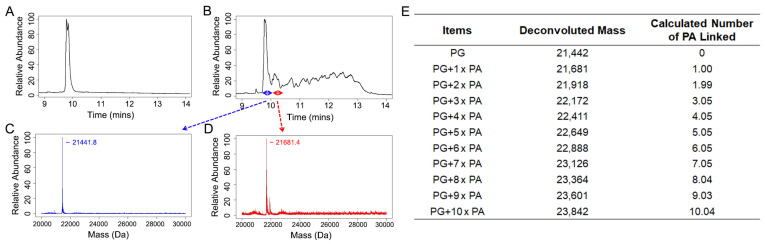
LC/QTOF/MS analysis of PA-PG. Chromatography profiles of PG (**A**) and PA-PG (**B**). Blue and red segments represent the first and second segments used for further deconvolution analysis. (**C**) Representative deconvoluted mass of the first segment. (**D**) Representative deconvoluted mass of the second segment. (**E**) Summary of deconvoluted mass and calculated number of PA molecules linked to one PG molecule.

PG was used to incorporate the targeting Ab on the surface of MSCs because PG binds to the Fc region of Ab and does not impact the antigen-binding complementarity-determining regions (CDRs). To localize PG on the cell membrane, PG was first derivatized with lipophilic PA. Insertion of PA into the cell membrane allows for extracellular localization of PG, which is then available to bind with the Ab [47]. We characterized PA-PG derivatives via LC/QTOF/MS. De-convolution of mass data showed successful conjugation of PA to PG and that up to ten molecules of PA can be added to each molecule of PG (Figure 1A–E).

**Figure 2:**
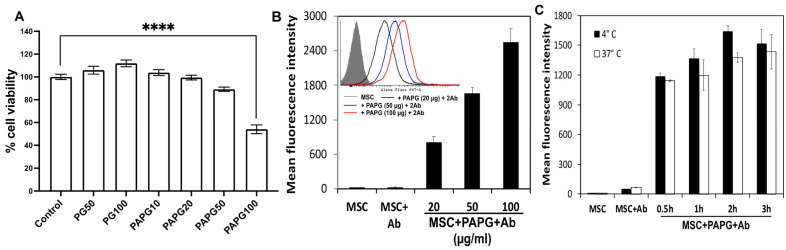
Optimization of PA-PG incorporation on MSC membrane. Effect of PA-PG concentration on cell viability (**A**) and Ab binding (**B**). The representative histogram overlay of different treatments is shown in the inset figure. Effect of incubation time and temperature on Ab binding (**C**). MSCs were treated with various concentrations of PA-PG for 1 h at 4 °C (**A**,**B**), or with 50 μg/mL of PA-PG for various time intervals at 4 °C or 37 °C (**C**). For (**B**,**C**), PA-PG-coated MSCs were treated with fluorescently labeled non-specific IgG. Untreated MSCs and MSCs treated with PG were used as controls. Cells were then analyzed via flow cytometry. **** *p* < 0.0001.

The effect of PA-PG incorporation on MSC viability was evaluated. Both PG and PA-PG had a minimal effect on cell viability at up to 50 μg/mL concentration (Figure 2A). We first optimized the concentration of PA-PG to successfully incorporate both PA-PG and the targeting Ab on MSC surface. PG (100 μg/mL) alone was used as a negative control. As shown in Figure 2A,B, the number of Ab molecules on the MSC surface increased with increasing concentration of PA-PG. Beyond 100 μg/mL concentration, PA-PG caused a significant loss of cell viability. We also evaluated the effect of time and temperature of the incubation reaction on PA-PG incorporation. As shown in Figure 2C, PA-PG incorporation increased with time and plateaued off beyond 2 h. There was not a significant effect of temperature on PA-PG incorporation. The lack of any effect of temperature suggested that PA-PG was bound physically to the cell surface and was not dependent on cellular energy.

**Figure 3:**
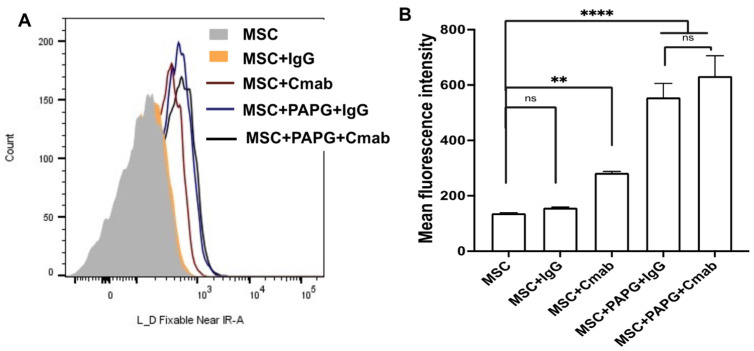
Antibody incorporation on PA-PG-coated MSCs. Coating of MSC surface with PA-PG was evaluated via flow cytometry. PA-PG-coated MSCs were treated with fluorescently labeled non-specific IgG or Cmab. Binding of IgG and Cmab to non-PA-PG treated MSCs were used as controls. (**A**) Representative histograms showing Ab binding for different groups and (**B**) the corresponding mean fluorescence intensity profiles. **** *p* < 0.0001; ** *p* < 0.01; ns = not significant.

**Figure 4:**
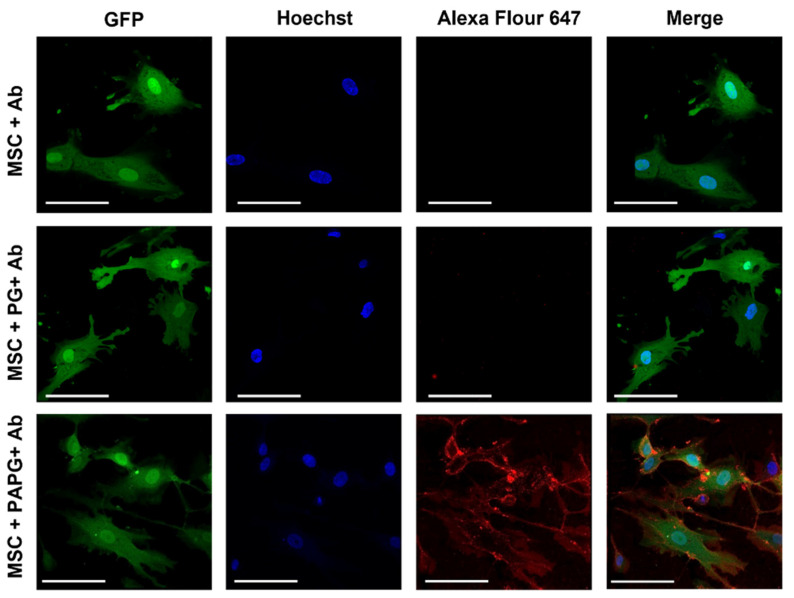
Visualization of labeled Ab-functionalized MSCs. GFP-expressing MSCs (green fluorescence) were treated with PA-PG and then with AF647-labeled Ab (red) for 1 h. Cells were counterstained with stained with Hoechst and then visualized using a confocal microscope for MSCs (green), nuclei (blue), and Ab (red) fluorescence. Overlay of images is shown in merged channel. Red fluorescence can be seen on the surface of cells. Scale bar: 20 µm.

The incorporation of the targeting Ab on MSCs was confirmed via flow cytometry. Figure 3A shows flow histograms following the incorporation of fluorescently labeled non-specific IgG, as well as anti-EGFR Ab, on PA-PG-coated MSCs compared to PG-coated MSCs and untreated MSCs. As shown in Figure 3B, an increase in fluorescence was observed for PA-PG-coated MSCs compared to the controls, demonstrating successful incorporation of PA-PG handle and its ability to bind with labeled Ab. There was no significant difference in the amount of Ab incorporated between the isotype IgG control or Cmab groups. We used confocal microscopy to confirm the presence of Ab on the surface of MSCs. As shown in Figure 4, the presence of labeled Ab (red fluorescence) could be clearly visualized on the surface of MSCs. We also determined the concentration of Ab bound to PA-PG-coated MSCs using fluorescence spectroscopy. These studies showed that there was ~1.75 μg Ab/10^6^ MSCs (~7 × 10^6^ Ab molecules/cell).

**Figure 5:**
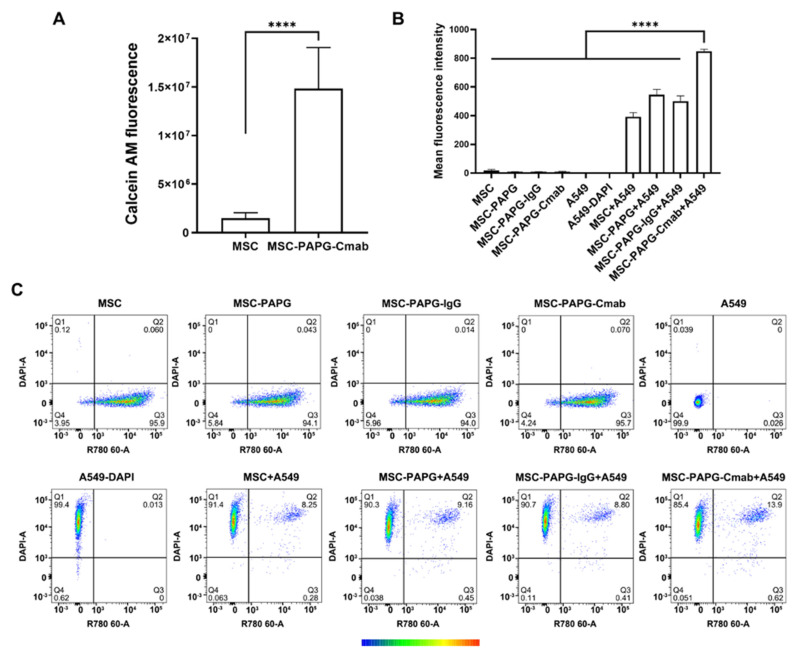
In vitro binding of EGFR-targeted MSCs to immobilize EGFR protein and EGFR-expressing A549 lung tumor cells. Binding of calcein AM stained unmodified MSCs and Cmab-MSCs to EGFR protein was measured via fluorescence spectroscopy (**A**). Flow cytometry was used to determine binding of AF647−labeled MSCs to Hoechst-stained A549 cells. Mean fluorescence intensity for the different groups is shown in (**B**). Individual dot plots are shown in (**C**). The color scale represents the density of events: blue (low) to red (high). **** *p* < 0.0001.

We then investigated the binding of Cmab-functionalized MSCs to their target. We initially evaluated the binding of MSCs to immobilized EGFR protein. As can be seen from Figure 5A, a greater number of Cmab-MSCs (as determined by calcein AM fluorescence) adhered to EGFR-protein-coated wells than non-targeted MSCs. We then investigated the binding of MSCs to EGFR-expressing A549 cells. While non-targeted MSCs (MSCs, MSCs coated with PA-PG, and MSCs incorporating non-specific control IgG) demonstrated some binding to A549 cells, Cmab-MSCs resulted in significantly greater binding to A549 cells (Figure 5B). These results provide evidence for the ability of targeting Ab incorporated on the surface of MSCs to recognize their intended target. PG binds all human subclasses of IgG with high affinity (K_D_ ~2 × 10^−10^ M) [48]. The K_D_ for Cmab binding to EGFR, on the other hand, is 3.8–11.0 × 10^−10^ M [49]. Cmab affinity for the protein expressed on the surface of cells is likely lower than for pure protein. The 2–3-fold (if not higher) difference in binding affinity for PG compared to that for EGFR could explain how MSCs retain the targeting Ab on the surface even in the presence of the target protein.

We next evaluated loading of paclitaxel in Cmab-MSCs. In previous studies, we demonstrated different approaches to stably load paclitaxel in MSCs [14,15,18,29]. These included simple endocytic loading by incubating MSCs with paclitaxel-loaded polymeric nanoparticles [29] or conjugating paclitaxel-loaded polymeric nanoparticles to the surface of MSCs [18]. Although the surface conjugation technique results in greater drug loading in MSCs, this approach could interfere with the incorporation of Cmab on the cell membrane. Hence, in the current study, we loaded the drug via simple incubation of MSCs with paclitaxel-loaded nanoparticles.

**Figure 6:**
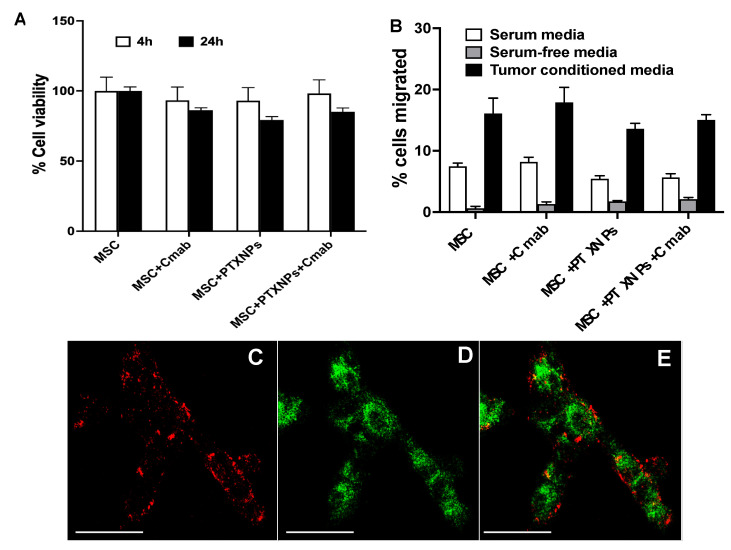
Paclitaxel loading in EGFR-targeted MSCs. Effect of paclitaxel loading on cell viability (**A**) and migration (**B**) of Cmab-MSCs (MSC + PTX NPs + Cmab). Controls included non-drug-loaded MSCs (MSC) and non-drug-loaded Cmab-MSCs (MSC + Cmab), or drug-loaded non-targeted MSCs (MSC + PTX NPs + IgG). Cell viability was measured via MTS assay after 4 and 24 h. Cell migration was determined using Transwell assay. (**C**–**E**) MSCs were engineered using 6-coumarin-labeled nanoparticles (green fluorescence), treated with PA-PG followed by incubation with AF647-labeled IgG (red fluorescence) for 1 h. Cells were visualized using a confocal microscope for (**C**) red and (**D**) green fluorescence. (**E**) shows overlay of the images from (**C**,**D**). Red fluorescence was on the surface while green fluorescence was intracellular. Scale bar: 20 µm.

Paclitaxel-loaded PLGA nanoparticles were prepared with a mean hydrodynamic diameter of ~350 nm with a negative zeta potential and drug loading of 20.3 ± 1.5% (*w*/*w*) and an entrapment efficiency of 76.2 ± 5.4%. As we reported previously, loading paclitaxel did not significantly affect the viability (Figure 6A) or migration (Figure 6B) of Cmab-MSCs when compared to non-drug-loaded MSCs and non-drug-loaded Cmab-MSCs, or drug-loaded non-targeted MSCs. We recently demonstrated that MSCs upregulate key antioxidant proteins such as Nrf2, superoxide dismutase, and heme oxygenase-1 in response to paclitaxel loading, which may explain how MSCs are resistant to high concentrations of paclitaxel. Other studies have shown that MSCs overexpress drug efflux transporters such as p-glycoprotein [29,50] and upregulate anti-apoptotic proteins [51], which may also contribute to drug resistance.

We next confirmed that loading paclitaxel in MSCs does not affect the surface localization of targeting Ab. Nanoparticles were labeled with green-fluorescent 6-coumarin while the Ab was labeled with red-fluorescent AF647. Confocal microscopy images demonstrated that Ab-associated red fluorescence was present on the MSC surface, while nanoparticle-associated green fluorescence was intracellular (Figure 6C–E). The distribution pattern of red fluorescence seen in this study was qualitatively similar to that seen in the case of non-drug-loaded MSCs (Figure 4), suggesting that drug loading does not affect Ab localization on MSC surface.

**Figure 7:**
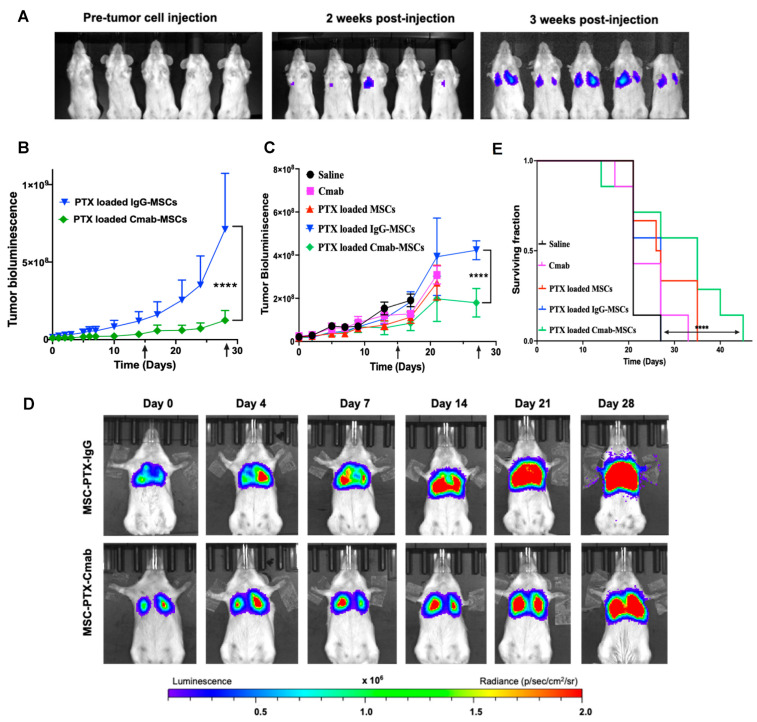
Antitumor efficacy of EGFR-targeted MSCs loaded with paclitaxel nanoparticles (PTX NP). (**A**) A549 lung tumors were generated using intravenous (tail vein) injection of A549-luc cells. Representative bioluminescence images showing lung tumor progression in mice following intravenous injection of A549-luc cells. (**B**) As a preliminary study, mice bearing A549 lung tumors were injected IV with 1 × 10^6^ IgG-MSCs (MSC + PTX NPs + IgG) or Cmab-MSCs (MSC + PTX NPs + Cmab) loaded with PTX NP (equivalent to 0.5 mg/kg PTX) every two weeks (arrows). Mice were imaged for tumor bioluminescence. (**C**) A full study was performed similar to (**B**) to include additional controls—saline, Cmab (equivalent concentration; 55 µg/kg), and 1 × 10^6^ MSCs loaded with PTX NP (equivalent to 0.5 mg/kg PTX) (MSC + PTX NPs). (**D**) Representative bioluminescence images of tumor growth at periodic time intervals post-treatment. (**E**) Mice were also monitored for survival. **** *p* < 0.0001 (n = 7).

We previously reported tumor-targeted delivery of paclitaxel using MSCs to orthotopic lung and ovarian tumors, resulting in improved therapeutic efficacy [15,16,18,29]. In the current study, we first investigated the comparative therapeutic efficacy of paclitaxel-loaded Cmab-MSCs relative to that of paclitaxel-loaded non-targeted IgG-MSCs. As can be seen from Figure 7B, mice that received Cmab-MSCs demonstrated significantly slower tumor growth than those that received control IgG-MSCs. These initial results demonstrated the potential for improved tumor targeting with Cmab-MSCs. Based on these promising results, a larger study was conducted to include additional controls. Since Cmab has therapeutic efficacy as a single agent [52,53], we included Cmab alone as a control. As observed in our previous studies, non-targeted MSCs loaded with paclitaxel nanoparticles were efficacious in slowing tumor growth and improving the overall survival relative to that of other controls (Figure 7C). However, the greatest tumor growth inhibition was observed in mice that received paclitaxel-loaded Cmab-MSCs. Compared to a median survival of 21 days for the saline-treated group and 27 days for the paclitaxel-loaded non-functionalized MSC-treated group, paclitaxel-loaded Cmab-MSC treatment resulted in a median survival of 35 days (*p* < 0.0001) (Figure 7E).

**Figure 8:**
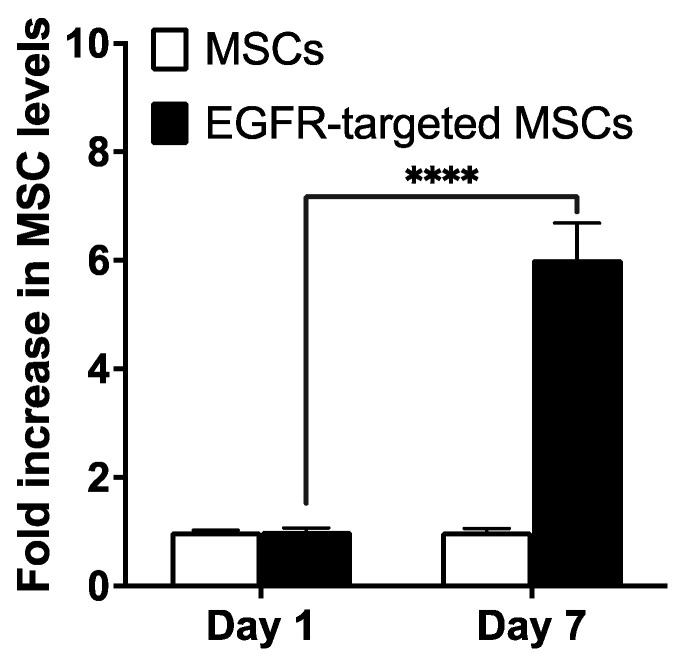
Tumor retention of EGFR-targeted MSCs. MSCs surface functionalized with anti-EGFR D8 Ab were injected intravenously in LL/2 lung-tumor-bearing mice. MSC levels in the lungs were quantified on days 1 and 7 by measuring mRNA levels of CD90, an MSC-specific protein. RPLP0 was used as the housekeeping gene. **** *p* < 0.0001 (n = 4).

We next evaluated the tumor-targeting effectiveness of EGFR-targeted MSCs. To fully understand the impact of a functional immune system on in vivo disposition of EGFR-targeted MSCs, the syngeneic, LL/2 lung tumor model was used. Tumors were induced by i.v. injection of LL/2-luc cells in immunocompetent C57/Bl6J mice. Further, since the goal was to evaluate the tumor accumulation of MSCs, we used RT-PCR to quantify human CD90 mRNA, which is specific to human MSCs and can be used to specifically and accurately quantify the MSC levels in the mouse tissue [54,55]. It has been previously shown that owing to the differential vascularity of organs, intravenously administered MSCs in healthy mice first travel to the lung, followed by the liver and spleen. However, the migration pattern and final residence could be different in the presence of different tumors and sites of inflammation [33,56]. There was no difference in MSC levels between non-targeted and targeted MSCs one day post-injection. However, there was a six-fold higher level of EGFR-targeted MSCs compared to non-targeted MSCs at 7 days post-injection (Figure 8). These results support the hypothesis that ligand functionalization will enhance MSC accumulation and retention in the tumor tissue.

Tumor homing of MSCs involves tethering of MSCs (mediated by CD44) to selectins expressed on endothelial cells followed by rolling on the vascular wall [57]. G-protein-coupled chemokine receptors (especially CXCR 4 and 7) activate MSCs in response to the expression of SDF-1 [58]. The activation of integrin α4β1 on MSCs in response to chemokines such as SDF-1 and subsequent binding to VCAM-1 on endothelial cells is a critical mechanism that facilitates the arrest and extravasation of MSCs in the tumor microenvironment, enabling their tumor-targeted delivery and therapeutic potential [59,60]. Researchers have explored various strategies to enhance the homing of MSCs to the tumor tissue, including overexpression of CXCR4 [43], magnetic targeting [35,57], and modulation of MSC surface to secrete proinflammatory cytokines [61]. The baseline expression of various cell surface proteins required for effective homing and arrest has been shown to be low (~10–13%) [62,63], which could explain the low efficiency of tumor accumulation with native MSCs.

We advance here a novel approach of incorporating tumor-targeting ligands on the MSC surface that allows for enhanced arrest and binding within the tumor tissue. Ko et al. previously demonstrated a surface coating method to target MSCs to inflammatory bowel disease (IBD) [47,64]. MSCs were coated with Abs against addressins to improve their colon delivery for treating Crohn’s disease and ulcerative colitis. Ab-coated MSCs showed improved treatment efficacy as evidenced by body weight and colon length recovery along with increased survival and immune cell suppression [64]. There are currently no reports on using ligand-functionalized MSCs for improved tumor targeting. Notably, the surface functionalization approach is non-genetic and highly modular. Thus, this technology can be used to incorporate any IgG, and can therefore be used to treat other tumor types by utilizing specific Abs against other tumor antigens such as HER2 or EphA2.

## 4. Conclusions

Our results demonstrate that incorporating the EGFR-targeting Ab Cmab improved MSC binding to immobilized EGFR protein and to tumor cells overexpressing EGFR. Further, Cmab-MSCs carrying paclitaxel resulted in a significantly improved antitumor effect in lung tumor-bearing mice. Cmab functionalization improved MSC accumulation and retention in a syngeneic mouse lung tumor model. Based on these results, we conclude that ligand functionalization could be used to enhance the concentration of therapeutic MSC constructs at the tumor tissue and to achieve improved antitumor response.

## Data Availability

The data that support the findings of this study will be made available by the corresponding author, SP, upon reasonable request.

## Abbreviations

MSCs	Mesenchymal stem cells
CAR	Chimeric antigen receptor
SAR	Synthetic antigen receptor
PG	Recombinant protein G
EGFR	Epidermal growth factor receptor
NSCLC	Non-small-cell lung cancer
Cmab	Cetuximab
PLGA	Poly (DL-lactide–co–glycolide)
PA	Palmitic acid
NHS	N-hydroxysuccinimide
DPBS	Dulbecco’s phosphate-buffered saline
PA-PG	Palmitic-acid-derivatized protein G
UPLC-MS	Ultra-performance liquid chromatography–mass spectrometry
HPLC	High-performance liquid chromatography
IACUC	Institutional Animal Care and Use Committee
SCID	Severe combined immunodeficiency
IgG-MSCs (MSC + PTX NPs + IgG)	IgG-antibody-coated, paclitaxel-nanoparticle-loaded MSCs
Cmab-MSCs (MSC + PTX NPs + Cmab)	Cetuximab-coated, paclitaxel-nanoparticle-loaded MSCs
DMEM	Dulbecco’s modified Eagle’s medium
MTS	(3-(4,5-dimethylthiazol-2-yl)-5-(3-carboxymethoxyphenyl)-2-(4-sulfophenyl)-2H-tetrazolium)
LC/QTOF/MS	Liquid Chromatography/Quadrupole Time-of-Flight/Mass Spectrometry
RT-PCR	Reverse transcription–polymerase chain reaction
PMS	Phenazine methosulfate
CDRs	Complementarity-determining regions
AF647-IgG	Alexa Flour 647-labeled IgG
Cmab-MSCs	Cetuximab-modified MSCs
RPLP0	Ribosomal Protein Lateral Stalk Subunit P0
HER2	Human epidermal growth factor receptor 2
EphA2	Ephrin type-A receptor 2
IBD	Inflammatory bowel disease
mRNA	Messenger RNA
w/v	Weight/volume
FBS	Fetal bovine serum
NP	Nanoparticles
PTX	Paclitaxel
i.p.	Intraperitoneal
i.v.	Intravenous
Luc	Luciferase
Ab	Antibody
ex	Excitation
em	Emission
min	Minutes
h	Hour

## References

[1] Siemann D.W. (2011). The unique characteristics of tumor vasculature and preclinical evidence for its selective disruption by Tumor-Vascular Disrupting Agents. Cancer Treat Rev..

[2] Forster J.C., Harriss-Phillips W.M., Douglass M.J., Bezak E. (2017). A review of the development of tumor vasculature and its effects on the tumor microenvironment. Hypoxia.

[3] Goel S., Duda D.G., Xu L., Munn L.L., Boucher Y., Fukumura D., Jain R.K. (2011). Normalization of the vasculature for treatment of cancer and other diseases. Physiol. Rev..

[4] Stylianopoulos T., Martin J.D., Chauhan V.P., Jain S.R., Diop-Frimpong B., Bardeesy N., Smith B.L., Ferrone C.R., Hornicek F.J., Boucher Y. (2012). Causes, consequences, and remedies for growth-induced solid stress in murine and human tumors. Proc. Natl. Acad. Sci. USA.

[5] Dewhirst M.W., Secomb T.W. (2017). Transport of drugs from blood vessels to tumour tissue. Nat. Rev. Cancer.

[6] Nichols J.W., Sakurai Y., Harashima H., Bae Y.H. (2017). Nano-sized drug carriers: Extravasation, intratumoral distribution, and their modeling. J. Control. Release.

[7] Tan Q., Saggar J.K., Yu M., Wang M., Tannock I.F. (2015). Mechanisms of Drug Resistance Related to the Microenvironment of Solid Tumors and Possible Strategies to Inhibit Them. Cancer J..

[8] Agrahari V., Agrahari V., Mitra A.K. (2017). Next generation drug delivery: Circulatory cells-mediated nanotherapeutic approaches. Expert Opin. Drug Deliv..

[9] Tan S., Wu T., Zhang D., Zhang Z. (2015). Cell or Cell Membrane-Based Drug Delivery Systems. Theranostics.

[10] Villa C.H., Anselmo A.C., Mitragotri S., Muzykantov V. (2016). Red blood cells: Supercarriers for drugs, biologicals, and nanoparticles and inspiration for advanced delivery systems. Adv. Drug Deliv. Rev..

[11] Huang B., Abraham W.D., Zheng Y., Bustamante López S.C., Luo S.S., Irvine D.J. (2015). Active targeting of chemotherapy to disseminated tumors using nanoparticle-carrying T cells. Sci. Transl. Med..

[12] Pang L., Qin J., Han L., Zhao W., Liang J., Xie Z., Yang P., Wang J. (2016). Exploiting macrophages as targeted carrier to guide nanoparticles into glioma. Oncotarget.

[13] Stuckey D.W., Shah K. (2014). Stem cell-based therapies for cancer treatment: Separating hope from hype. Nat. Rev. Cancer.

[14] Layek B., Sadhukha T., Panyam J., Prabha S. (2018). Nano-Engineered Mesenchymal Stem Cells Increase Therapeutic Efficacy of Anticancer Drug Through True Active Tumor Targeting. Mol. Cancer Ther..

[15] Layek B., Sadhukha T., Prabha S. (2016). Glycoengineered mesenchymal stem cells as an enabling platform for two-step targeting of solid tumors. Biomaterials.

[16] Layek B., Shetty M., Nethi S.K., Sehgal D., Starr T.K., Prabha S. (2020). Mesenchymal Stem Cells As Guideposts for Nanoparticle-Mediated Targeted Drug Delivery in Ovarian Cancer. Cancers.

[17] Cheng S., Nethi S.K., Al-Kofahi M., Prabha S. (2021). Pharmacokinetic—Pharmacodynamic Modeling of Tumor Targeted Drug Delivery Using Nano-Engineered Mesenchymal Stem Cells. Pharmaceutics.

[18] Layek B., Sehgal D., Argenta P.A., Panyam J., Prabha S. (2019). Nanoengineering of Mesenchymal Stem Cells via Surface Modification for Efficient Cancer Therapy. Adv. Ther..

[19] Turtle C.J., Hanafi L.-A., Berger C., Gooley T.A., Cherian S., Hudecek M., Sommermeyer D., Melville K., Pender B., Budiarto T.M. (2016). CD19 CAR–T cells of defined CD4+:CD8+ composition in adult B cell ALL patients. J. Clin. Investig..

[20] Miliotou N.A., Papadopoulou C.L. (2018). CAR T-cell Therapy: A New Era in Cancer Immunotherapy. Curr. Pharm. Biotechnol..

[21] Dennis J.E., Cohen N., Goldberg V.M., Caplan A.I. (2004). Targeted delivery of progenitor cells for cartilage repair. J. Orthop. Res..

[22] Choe W., Durgannavar T.A., Chung S.J. (2016). Fc-Binding Ligands of Immunoglobulin G: An Overview of High Affinity Proteins and Peptides. Materials.

[23] Inamura K., Ninomiya H., Ishikawa Y., Matsubara O. (2010). Is the Epidermal Growth Factor Receptor Status in Lung Cancers Reflected in Clinicopathologic Features?. Arch. Pathol. Lab. Med..

[24] Salimath S., Jayaraj B.S., Mahesh P.A. (2015). Epidermal growth factor receptor (EGFR) expression in non-small cell lung carcinoma (NSCLC) and survival. Eur. Respir. J..

[25] Masuda H., Zhang D., Bartholomeusz C., Doihara H., Hortobagyi G.N., Ueno N.T. (2012). Role of epidermal growth factor receptor in breast cancer. Breast Cancer Res. Treat.

[26] Gatzemeier U., von Pawel J., Vynnychenko I., Zatloukal P., de Marinis F., Eberhardt W.E.E., Paz-Ares L., Schumacher K.-M., Goddemeier T., O’Byrne K.J. (2011). First-cycle rash and survival in patients with advanced non-small-cell lung cancer receiving cetuximab in combination with first-line chemotherapy: A subgroup analysis of data from the FLEX phase 3 study. Lancet Oncol..

[27] Lim S.I., Mizuta Y., Takasu A., Hahn Y.S., Kim Y.H., Kwon I. (2013). Site-specific fatty acid-conjugation to prolong protein half-life in vivo. J. Control Release.

[28] Ross K., Tu T., Smith S., Dalluge J. (2007). Profiling of Organic Acids during Fermentation by Ultraperformance Liquid Chromatography−Tandem Mass Spectrometry. Anal. Chem..

[29] Sadhukha T., O’Brien T.D., Prabha S. (2014). Nano-engineered mesenchymal stem cells as targeted therapeutic carriers. J. Control. Release.

[30] Prabha S., Merali C., Sehgal D., Nicolas E., Bhaskar N., Flores M., Bhatnagar S., Nethi S.K., Barrero C.A., Merali S. (2023). Incorporation of paclitaxel in mesenchymal stem cells using nanoengineering upregulates antioxidant response, CXCR4 expression and enhances tumor homing. Mater Today Bio.

[31] Ullah I., Subbarao R.B., Rho G.J. (2015). Human mesenchymal stem cells-current trends and future prospective. Biosci. Rep..

[32] Zhang X., Hirai M., Cantero S., Ciubotariu R., Dobrila L., Hirsh A., Igura K., Satoh H., Yokomi I., Nishimura T. (2011). Isolation and characterization of mesenchymal stem cells from human umbilical cord blood: Reevaluation of critical factors for successful isolation and high ability to proliferate and differentiate to chondrocytes as compared to mesenchymal stem cells from bone marrow and adipose tissue. J. Cell Biochem..

[33] El Marsafy S., Larghero J. (2015). Mesenchymal Stem Cells: Key Actors in Tumor Niche. Curr. Stem Cell Res. Ther..

[34] Cheng S., Nethi S.K., Rathi S., Layek B., Prabha S. (2019). Engineered Mesenchymal Stem Cells for Targeting Solid Tumors: Therapeutic Potential beyond Regenerative Therapy. J. Pharm. Exp..

[35] De Becker A., Riet I.V. (2016). Homing and migration of mesenchymal stromal cells: How to improve the efficacy of cell therapy?. World J. Stem. Cells.

[36] Liu H., Liu S., Li Y., Wang X., Xue W., Ge G., Luo X. (2012). The role of SDF-1-CXCR4/CXCR7 axis in the therapeutic effects of hypoxia-preconditioned mesenchymal stem cells for renal ischemia/reperfusion injury. PLoS ONE.

[37] Guan J., Chen J. (2013). Mesenchymal stem cells in the tumor microenvironment. Biomed. Rep..

[38] Razeghian E., Margiana R., Chupradit S., Bokov D.O., Abdelbasset W.K., Marofi F., Shariatzadeh S., Tosan F., Jarahian M. (2021). Mesenchymal Stem/Stromal Cells as a Vehicle for Cytokine Delivery: An Emerging Approach for Tumor Immunotherapy. Front. Med..

[39] Niess H., Bao Q., Conrad C., Zischek C., Notohamiprodjo M., Schwab F., Schwarz B., Huss R., Jauch K.W., Nelson P.J. (2011). Selective targeting of genetically engineered mesenchymal stem cells to tumor stroma microenvironments using tissue-specific suicide gene expression suppresses growth of hepatocellular carcinoma. Ann. Surg..

[40] Song C., Xiang J., Tang J., Hirst D.G., Zhou J., Chan K.-M., Li G. (2011). Thymidine Kinase Gene Modified Bone Marrow Mesenchymal Stem Cells as Vehicles for Antitumor Therapy. Hum. Gene Ther..

[41] Yin J., Kim J.-K., Moon J.-H., Beck S., Piao D., Jin X., Kim S.-H., Lim Y.C., Nam D.-H., You S. (2011). hMSC-mediated Concurrent Delivery of Endostatin and Carboxylesterase to Mouse Xenografts Suppresses Glioma Initiation and Recurrence. Mol. Ther..

[42] Balyasnikova I.V., Ferguson S.D., Sengupta S., Han Y., Lesniak M.S. (2010). Mesenchymal stem cells modified with a single-chain antibody against EGFRvIII successfully inhibit the growth of human xenograft malignant glioma. PLoS ONE.

[43] Wang J., Zhang W., He G.H., Wu B., Chen S. (2018). Transfection with CXCR4 potentiates homing of mesenchymal stem cells in vitro and therapy of diabetic retinopathy in vivo. Int. J. Ophthalmol..

[44] Moku G., Layek B., Trautman L., Putnam S., Panyam J., Prabha S. (2019). Improving Payload Capacity and Anti-Tumor Efficacy of Mesenchymal Stem Cells Using TAT Peptide Functionalized Polymeric Nanoparticles. Cancers.

[45] Wang X., Gao J., Ouyang X., Wang J., Sun X., Lv Y. (2018). Mesenchymal stem cells loaded with paclitaxel-poly(lactic-co-glycolic acid) nanoparticles for glioma-targeting therapy. Int. J. Nanomed..

[46] Yang C.H., Chou H.C., Fu Y.N., Yeh C.L., Cheng H.W., Chang I.C., Liu K.J., Chang G.C., Tsai T.F., Tsai S.F. (2015). EGFR over-expression in non-small cell lung cancers harboring EGFR mutations is associated with marked down-regulation of CD82. Biochim. Biophys. Acta.

[47] Ko I.K., Kean T.J., Dennis J.E. (2009). Targeting mesenchymal stem cells to activated endothelial cells. Biomaterials.

[48] Rispens T., Vidarsson G., Ackerman M.E., Nimmerjahn F. (2014). Chapter 9-Human IgG Subclasses. Antibody Fc.

[49] Patel D., Lahiji A., Patel S., Franklin M., Jimenez X., Hicklin D.J., Kang X. (2007). Monoclonal antibody cetuximab binds to and down-regulates constitutively activated epidermal growth factor receptor vIII on the cell surface. Anticancer Res..

[50] Dai T., Yang E., Sun Y., Zhang L., Zhang L., Shen N., Li S., Liu L., Xie Y., Wu S. (2013). Preparation and drug release mechanism of CTS-TAX-NP-MSCs drug delivery system. Int. J. Pharm..

[51] Khubutiya M.S., Vagabov A.V., Temnov A.A., Sklifas A.N. (2014). Paracrine mechanisms of proliferative, anti-apoptotic and anti-inflammatory effects of mesenchymal stromal cells in models of acute organ injury. Cytotherapy.

[52] Yang J., Mo J., Dai J., Ye C., Cen W., Zheng X., Jiang L., Ye L. (2021). Cetuximab promotes RSL3-induced ferroptosis by suppressing the Nrf2/HO-1 signalling pathway in KRAS mutant colorectal cancer. Cell Death Dis..

[53] Mazzarella L., Guida A., Curigliano G. (2018). Cetuximab for treating non-small cell lung cancer. Expert Opin. Biol..

[54] Michelis K.C., Nomura-Kitabayashi A., Lecce L., Franzén O., Koplev S., Xu Y., Santini M.P., D’Escamard V., Lee J.T.L., Fuster V. (2018). CD90 Identifies Adventitial Mesenchymal Progenitor Cells in Adult Human Medium- and Large-Sized Arteries. Stem. Cell Rep..

[55] L Ramos T., Sánchez-Abarca L.I., Muntión S., Preciado S., Puig N., López-Ruano G., Hernández-Hernández Á., Redondo A., Ortega R., Rodríguez C. (2016). MSC surface markers (CD44, CD73, and CD90) can identify human MSC-derived extracellular vesicles by conventional flow cytometry. Cell Commun. Signal..

[56] Kidd S., Spaeth E., Dembinski J.L., Dietrich M., Watson K., Klopp A., Battula V.L., Weil M., Andreeff M., Marini F.C. (2009). Direct Evidence of Mesenchymal Stem Cell Tropism for Tumor and Wounding Microenvironments Using In Vivo Bioluminescent Imaging. Stem. Cells.

[57] Ullah M., Liu D.D., Thakor A.S. (2019). Mesenchymal Stromal Cell Homing: Mechanisms and Strategies for Improvement. iScience.

[58] Lau T.T., Wang D.-A. (2011). Stromal cell-derived factor-1 (SDF-1): Homing factor for engineered regenerative medicine. Expert Opin. Biol. Ther..

[59] Cook-Mills J.M., Marchese M.E., Abdala-Valencia H. (2011). Vascular cell adhesion molecule-1 expression and signaling during disease: Regulation by reactive oxygen species and antioxidants. Antioxid. Redox Signal.

[60] Kavanagh D.P., Durant L.E., Crosby H.A., Lalor P.F., Frampton J., Adams D.H., Kalia N. (2010). Haematopoietic stem cell recruitment to injured murine liver sinusoids depends on (alpha)4(beta)1 integrin/VCAM-1 interactions. Gut.

[61] Liu H., Zhu X., Cao X., Chi A., Dai J., Wang Z., Deng C., Zhang M. (2021). IL-1β-primed mesenchymal stromal cells exert enhanced therapeutic effects to alleviate Chronic Prostatitis/Chronic Pelvic Pain Syndrome through systemic immunity. Stem Cell Res..

[62] Honczarenko M., Le Y., Swierkowski M., Ghiran I., Glodek A.M., Silberstein L.E. (2009). Human Bone Marrow Stromal Cells Express a Distinct Set of Biologically Functional Chemokine Receptors. Stem Cells.

[63] Ponte A.L., Marais E., Gallay N., Langonné A., Delorme B., Hérault O., Charbord P., Domenech J. (2007). The In Vitro Migration Capacity of Human Bone Marrow Mesenchymal Stem Cells: Comparison of Chemokine and Growth Factor Chemotactic Activities. Stem Cells.

[64] Ko I.K., Kim B.-G., Awadallah A., Mikulan J., Lin P., Letterio J.J., Dennis J.E. (2010). Targeting Improves MSC Treatment of Inflammatory Bowel Disease. Mol. Ther..

